# Immunohistochemical Expression of CXCR4 on Breast Cancer and Its Clinical Significance

**DOI:** 10.1155/2015/891020

**Published:** 2015-06-16

**Authors:** Marina Okuyama Kishima, Carlos Eduardo Coral de Oliveira, Bruna Karina Banin-Hirata, Roberta Losi-Guembarovski, Karen Brajão de Oliveira, Marla Karine Amarante, Maria Angelica Ehara Watanabe

**Affiliations:** ^1^Laboratory of Human Pathology, Department of Pathology, Clinical Analysis and Toxicology, Health Sciences Center, University Hospital of Londrina, State University of Londrina, 86038-350 Londrina, PR, Brazil; ^2^Laboratory of Study and Application of DNA Polymorphisms and Immunology, Department of Pathological Sciences, Biological Sciences Center, State University of Londrina, 86057-970 Londrina, PR, Brazil; ^3^Laboratory of Molecular Genetics and Immunology, Department of Pathological Sciences, Biological Sciences Center, State University of Londrina, 86057-970 Londrina, PR, Brazil

## Abstract

Many tumor cells express chemokines and chemokine receptors, and, for this reason, these molecules can affect the tumor progression. It is known that breast cancer is a complex and heterogeneous neoplasia comprising distinct diseases, histological characteristics, and clinical outcomes. The most studied role for CXCL12 chemokine and its receptor CXCR4 in breast cancer pathogenesis is the metastasis event, although several reports have demonstrated its involvement in other processes, such as angiogenesis and tumor growth. It has been found that CXCR4 is required for breast cancer cell migration to other sites such as lung, bone, and lymph nodes, which express high levels of CXCL12 chemokine. Therefore, CXCR4 is being considered a prognostic marker in breast cancer. Within this context, this review summarizes established studies involving expression of CXCR4 on breast cancer, focusing on its clinical significance.

## 1. Introduction

Chemokines are potent chemotactic molecules involved not only in inflammation processes and immune surveillance but also in important physiological and pathological conditions, such as cancer progression [1]. Some data have suggested that the chemokine receptor network could be involved in the seed and soil phenomenon and that metastasis could be localized according to chemokine receptor expression [2].

Many retrospective studies have documented the expression of various chemokine receptors, particularly the C-X-C chemokine receptor 4 (CXCR4), and the association with higher recurrence incidences and cancer-related deaths, tumor size, advanced tumor-node-metastasis (TNM) stage, and shorter survival, predicting poor prognosis to cancer patients [3, 4].

Among different types of malignant tumours, breast cancer is the most common among women, in both developed and developing countries, with approximately 1,7 million new cases and 560,000 deaths worldwide every year [5, 6]. During the last decade, researches have focused deeply on the molecular biology of this disease. Technological breakthroughs and in particular high throughput approaches have allowed researchers to inquire into the nature of breast cancer, uncovering intrinsic and orchestrated interconnection of several signaling pathways, and the cellular microenvironment influencing disease pathophysiology, outcome, and treatment response [7].

There are opposing reports in the literature regarding the expression of CXCR4 in breast cancer stating that it is increased, decreased, or not changed compared to benign epithelium. Furthermore, there is disagreement amongst the authors with regard to CXCR4 localization in malignant cells: some describe peripheral or nuclear staining, while the majority states that CXCR4 expression is predominantly cytoplasmic.

The tumoral CXCR4 expression has been evaluated by several methods, one of which being commonly used is the standard immunohistochemical staining. Published data demonstrated the analysis of CXCR4 expression by immunohistochemistry in breast cancer tissue and cells and its clinical significance regarding the localization and expression patterns, emphasizing its distinguished relevance as a prognostic marker. Inside this context, this review focused on reporting the importance of immunohistochemical expression of CXCR4 on breast cancer and its clinical significance.

## 2. CXCR4 and Breast Cancer

CXCR4 is a rhodopsin-like G-protein coupled receptor (GPCR), displaying 7-transmembrane helical domains, involved in important signaling functions. The GPCRs signaling pathway has been discussed in a review by Cojoc et al. [8]. Briefly, upon ligand interaction and receptor activation, the GPCR promotes conformation changes within the trimeric G protein (G*α*/G*β*/G*γ*), releasing from the receptor the G*α* and G*βγ* subunits, which interact with various effector proteins and initiate intracellular signaling cascades, leading to several processes, such as stemness, survival, proliferation, and chemotaxis.

The CXCL12 chemokine and its receptor CXCR4 are molecules with important immunological functions, which have further relevant roles in the context of mammary carcinogenesis. Thus, the significance of the CXCL12/CXCR4 axis in breast cancer invasion and metastasis has been widely investigated [9–11].

CC and CXC chemokine receptor expression is described on a wide variety of cancer cell types [12–14], including at least 23 different hematopoietic, epithelial, and mesenchymal tumours [12]. On the other hand, within primary tumours and cancer cell lines, for example, ovarian and non-small-cell lung cancer, only a subpopulation of cells expresses the CXCR4 receptor [15].

In human cells, CXCR4 expression was detected in many cells, for instance, bronchial epithelial cells [16], endothelial cells [17, 18], fibrocytes [19], lymphocytes [20], intestinal (including colonic) epithelial cells [21, 22], microglia, neurons, and astrocytes [23], primitive hematopoietic progenitor cells [24], vascular smooth muscle cells [25], and pluripotent stem cells, including mammary stem cells [26]. Further, according to Müller et al. [27], CXCR4 is present at a low level or even absent in normal breast tissue but is highly expressed in both primary and metastatic breast tumours.

Particularly, CXCR4 expression on breast tumor cells can be regulated by several factors, such as hypoxia, vascular endothelial growth factor (VEGF), nuclear factor kappa-light-chain-enhancer of activated B cells (NF-*κ*B), estrogen, transforming growth factor-beta 1 (TGF-*β*1), and IFN-*γ*, which have been shown to upregulate this receptor in tumor microenvironment [28–30]. Furthermore, its expression can be regulated by epigenetic mechanisms, like cytosine methylation, whose pattern was correlated with clinicopathological aspects of primary breast tumours, in a study developed by Ramos et al. [31].

As peripheral lymphocytes preferentially localize to peripheral lymphoid tissues, such as lymph nodes, chemokine receptors in tumoral cells drive invasion and metastization to specific sites. Indeed, CXCR4 is overexpressed in metastatic breast cancer cells; consequently it critically mediates the homing process to specific metastatic sites [27]. Notably, this is the most devastating process attributed to cancer and significantly influences its morbidity and mortality [32].

The first steps of metastasis are characterized by increased motility and invasiveness, and it has been hypothesized that they are associated with the epithelial-mesenchymal transition (EMT), a process by which epithelial cells acquire mesenchymal fibroblast-like properties and show reduced intercellular adhesion and increased motility [33]. Notwithstanding the CXCR4 involvement in chemotaxis process, CXCR4 can drive epithelial to mesenchymal transition along with an upregulation of other chemokine receptors and cytokines, leading to cell migration, lymphatic invasion, and tumor metastasis [34].

## 3. Immunohistochemical Expression of CXCR4 on Breast Cancer 

Application of immunohistochemistry in clinical laboratory has significantly contributed to the diagnostic and stratification of many cancer types, particularly breast cancer. The detection of both estrogen and progesterone receptors (ERs and PRs) is strongly predictive for endocrine therapy response but weak prognostic markers of clinical outcome in breast cancer [35]. Therefore, the detection of many other markers, including CXCR4, allows better evaluation of breast cancer, which can strongly influence the therapy conduct (guidance) and survival among patients.

In this particular aspect, important factors may affect IHC efficiency and reproducibility, not to mention tissue fixation (both type and duration), the choice of antibody, and the threshold for interpretation of positive immunostaining [35]. This may explain, in part, why there are controversial data regarding CXCR4 staining in different studies. Nonetheless, caution in evaluating the associative results regarding both CXCR4 and CXCL12 expressions in the tumor microenvironment is highly recommended.

In a series of elegant IHC studies with anti-human CXCR4 monoclonal antibody, CXCR4 positivity was assessed semiquantitatively using staining intensity and percentage. Li et al. [36] demonstrated that the human epithelial growth factor receptor 2 (HER2, ErbB2, and neu), which is amplified or overexpressed in about 30% of breast cancers and is a known marker of poor prognosis, enhanced CXCR4 expression and this was required for HER2-induced lung metastasis.

Kato et al. [37] examined the expression of CXCR4 protein in breast tumours, which was significantly correlated with metastatic potential of primarily involved lymph nodes to secondary nodes. Initially, the authors suggested that the majority of breast tumors have cells with different CXCR4 expression levels, and this was directly related to the metastatic potential. However, this study also revealed that breast cancer cells express CXCR4 protein diffusely but at equal levels and that heterogeneity was rarely observed by immunohistochemical staining. Conversely, some tumours showed heterogenous CXCR4 focal staining, which was clearly different from other cases with CXCR4 diffuse staining.

Bohn et al. [38] described a qualitatively (positive or negative) staining pattern of breast cancer using a rabbit polyclonal (1 : 100) antibody for human CXCR4 (cytoplasm and membrane). They did not find differences between nuclear and cytoplasmic CXCR4 expressions in primary and bone metastasis of breast cancer, and nearly all tumours showed strong immunoreactivity. Possible explanations to these findings could be related to the specific molecular mechanisms responsible for nodal metastasis, the intrinsic tumor biology, and the differences related to the antibody.

It has been suggested that CXCR4 expression in primary tumor is associated with a higher risk for bone metastasis [39]. Using anti-human CXCR4 monoclonal antibody, the immunoreactivity of membrane and cytoplasm was evaluated in breast cancer and correlated to sites of metastasis, particularly in CXCL12-producing organs. Although CXCR4 expression was not associated with clinical characteristics, prognostic for overall survival and higher rate of liver, lung, and brain metastasis, CXCR4-positive tumours showed a significantly higher risk for bone metastasis, opening new perspectives for the development of novel adjuvant strategies targeting bone tissue.

According to these findings, Sun et al. [40] have reported that CXCL12-CXCR4 axis correlated tightly with breast cancer metastasis. In this work, membrane and cytoplasmic CXCR4 staining was evaluated by IHC. Further, CXCR4 expression was significantly associated with lymph node metastasis and TNM stage. In contrast, van den Berg et al. [41] have shown that CXCR4 labeling was predominantly on the cell membrane and/or in vesicles formed after endocytosis, using confocal imaging. Deparaffinized MDAMB231 tumor tissue incubated with Ac-TZ14011–FITC showed strong nonspecific staining in the nuclear membrane, nucleoli, and connective tissue. However, MDAMB231^CXCR4+^ tumor tissue slides showed somewhat different staining profile, with cytoplasmic and membrane predominant staining.

It is already known that the GPCRs, such as CXCR4, also undergo internalization upon interaction with their respective ligand. In particular, the endocytosis induced by CXCL12 has been demonstrated in tumor cells [42]. And although this study confirmed the existence of a general regulatory mechanism of intracellular expression of the endocytosis extent, kinetics and recycling differ between cell types; it is reasonable that it has implications for traffic regulation and functional consequences [43, 44]. The predominant intracellular localization of CXCR4 suggests that the dynamic equilibrium between the plasma membrane and cytoplasm can modulate CXCR4 availability at the cell surface, which in turn regulates cell responsiveness to follow a CXCL12 concentration gradient.

In this context, Hassan et al. [45] hypothesized that tumours overexpressing CXCR4 have an enhanced ability to metastasize in patients with low CXCL12 plasma levels. The rate of breast cancer specific mortality was higher in patients with both high phosphorylated-CXCR4 expression and low plasma CXCL12 levels than either low plasma CXCL12 or high phosphorylated-CXCR4 expression alone. Using a biotin-labeled CXCR4 antagonist, TN14003, the IHC analysis of phosphorylated-CXCR4 and CXCR4 from the tissue microarray revealed cytoplasmic and nuclear expression for both biomarkers and highlighted the prognostic value of evaluating the phosphorylated-CXCR4 expression.

The expression level and cellular localization of CXCR4 in human breast tumours have also been analyzed in view of its molecular subtypes, including triple-negative breast cancers (TNBC), luminal subtypes, and HER2-positive breast cancers, via IHC. Chen et al. [46] found that TNBC expressed CXCR4 more frequently than other subtypes, and its expression was primarily detected in the cytoplasm of tumor cells using a semiquantitative scoring system. Their study also indicated that CXCR4-positive TNBC correlated with poor clinical prognosis and was consistent with the unique visceral metastasis pattern, suggesting an effective application in TNBC treatment.

Differently, Zhang et al. [47] detected the CXCR4 protein expression by IHC in other breast cancer subtypes. These authors detected higher expression levels of the receptor in basal-like subtype, while in luminal A the expression was lower. In accordance with their study, Sivrikoz et al. [48] demonstrated that, besides basal-like subtype, the HER2 enriched breast cancer subtype also expresses high CXCR4 protein compared to the other subtypes.

Furthermore, Chu et al. [49] found that basal-like tumor patients with high CXCR4 expression had significantly higher recurrence incidence and related death than those with low CXCR4 protein expression. Hence, higher CXCR4 expression in cancer specimens might predict a worse outcome in patients with basal-like breast cancer subtype.

One of the key points on describing the role of the CXCR4/CXCL12 axis in breast cancer pathogenesis is concerned with the IHC analysis of only CXCR4, once limited information on CXCL12 expression has been published. In this regard, Papatheodorou et al. [50] investigated the immunoexpression of CXCR4 and CXCL12 in invasive breast carcinomas and suggested that the differential CXCR4 expression at intratumoral stroma and CXCL12 in adjacent normal mammary cells may predict clinical outcome in breast cancer patients. High nuclear and cytoplasmic CXCR4 expression was detected in cancer cells. Differently, mammary epithelial cells in the adjacent nonneoplastic breast tissue showed low cytoplasmic and nuclear CXCR4 expression, in the vast majority of cases. Fibroblasts of normal stroma showed lower CXCR4 expression, while intratumoral fibroblasts were highly positive. In intratumoral endothelial cells, high CXCR4 expression was detected, while normal endothelium was negative in all cases. As far as expression patterns in normal breast tissue are concerned, results from Papatheodorou et al. [50] are in accordance with previous studies reporting overexpression of CXCR4 in breast carcinomas compared with normal breast parenchyma [37, 51–53]. Curiously, higher CXCR4 expression in intratumoral fibroblasts was positively correlated with survival rates of patients, and both CXCL12 and CXCR4 expressions were correlated in cancer cells, intratumoral fibroblasts, and endothelial cells.

Interestingly, Aravindan et al. [54] evaluated CXCR4 and CXCL12 immunoreactivity beside cytoplasm and observed, to a lesser extent, nuclei expression in tumour epithelial cells. CXCR4 showed cytoplasmic immunoreactivity in 38.2% of tumor samples and 9.1% in normal samples using primary antibodies specific for CXCR4.

Given the diversity of findings, the difficulty in evaluating published results from different samples to the complexity of breast cancer subtypes, and the limitations of IHC analysis patterns, it is difficult to draw definitive conclusions about the role of CXCR4 in breast malignancy. However, an apparent theme is the contrast between cellular CXCR4 localization through immunohistochemical staining in tumor cell lines and breast tissue and the correlation with a more aggressive clinical outcome. It is reasonable that the role of CXCR4 in breast tumors might involve more than positive and higher expression levels in tumoral cells.

Those differences regarding positivity and localization of CXCR4 expression in breast cancer and its subtypes may represent aberrant proportion of variants, somatic mutations, or different transcriptional or cotranslational modifications [55]. Because CXCR4 represents a physiologic target for CXCL12 interaction in normal cellular signaling, it is conceivable that certain cytoplasmic staining may indicate CXCR4 internalization in response to ligand binding [41, 56]. In these cases, increased cytoplasmic CXCR4 immunostaining might abrogate membrane expression, thus limiting tumor cell evasion, and, possibly, would be associated with nonmetastatic profile.

In fact, CXCL12 expression was associated with disease-free and overall survival in a highly significant manner in two independent microarray gene expression datasets as well as in 100 breast cancer cases, analysed by IHC [57]. Compelling evidences indicate the potential protumoral, but not necessarily prometastatic, activity for CXCL12, such as proliferation mediation, apoptosis inhibition, and angiogenesis induction [27, 58].

Another important point to consider is the functional status of immune subsets within the tumor microenvironment. Heterogenous cellular CXCR4 localization must suggest divergent biological singularities in each patient, once antitumor immune response could be correlated to degrees of host immunocompetence. There is little experimental data demonstrating CXCR4 expression status in different breast tumor microenvironments, considering the mediators of inflammation related to cancer. These can induce the CXCR4 increasing expression and, depending on the circumstances, other mediators can attenuate this expression [55].

Based on these studies we conclude that there is a complex interaction between CXCR4 expressed on tumor cells with CXCL12 present in the surrounding stroma. Importantly, it is noteworthy that there is also an immunological context in tumoral microenvironment, where often exists an infiltration of intratumoral or peritumoral immune cells also expressing CXCR4. Besides, simply quantifying the CXCR4 expression by IHC can lead to assumptions that do not evaluate the CXCR4/CXCL12 axis. Furthermore, assessment of gene expression or intracellular cytoplasmic CXCR4 detection by flow cytometry could complement the analysis by IHC and indicate how CXCR4 and CXCL12 are behaving at the molecular level, deciphering the interface of this axis in the breast cancer pathobiology.
